# Dataset of allele, genotype and haplotype frequencies of four *LIN28B* gene polymorphisms analyzed for association with age at menarche in Russian women

**DOI:** 10.1016/j.dib.2019.104323

**Published:** 2019-07-30

**Authors:** Irina Ponomarenko, Evgeny Reshetnikov, Oleg Golovchenko, Alexey Polonikov, Irina Verzilina, Inna Sorokina, Inna Aristova, Anna Yermachenko, Volodymyr Dvornyk, Mikhail Churnosov

**Affiliations:** a Department of Medical Biological Disciplines, Belgorod State University, 308015, Belgorod, Russia; b Department of Obstetrics and Gynecology, Belgorod State University, 308015, Belgorod, Russia; c Department of Biology, Medical Genetics and Ecology, Kursk State Medical University, 305041, Kursk, Russia; d Department of Social Epidemiology, Pierre Louis Institute of Epidemiology and Public Health, 75571, Paris, France; e Sorbonne Universités, 75320, Paris, France; f Department of Life Sciences, College of Science and General Studies, Alfaisal University, 11533, Riyadh, Saudi Arabia

**Keywords:** *LIN28B* gene, Single nucleotide polymorphism, Age at menarche

## Abstract

In this paper, we present the allele, genotype and haplotype frequencies of 4 single nucleotide polymorphisms (SNPs) in *LIN28B* gene (rs4946651, rs7759938, rs314280, rs314276) in a sample of Russian women. These SNPs had been previously identified to be associated with age at menarche in genome-wide association studies (GWAS). The information about age at menarche was obtained using the questionnaire. The frequencies of alleles, genotypes and haplotypes of four SNPs were classified in 3 groups: the whole sample, individuals with the early age at menarche (<12 years), and those with the average age at menarche (12–14 years).

**Table:** Specifications Table

Subject area	*Biology*
More specific subject area	*Genetics*
Type of data	*Table and figure*
How data was acquired	*MALDI/TOF mass spectrometry using Sequenom MassARRAY 4.0 platform (Agena Bioscience™)*
Data format	*Raw and analyzed data*
Experimental factors	*Total genomic DNA was isolated from buffy coat using the standard phenol-chloroform method.*
Experimental features	*DNA samples were genotyped using the Sequenom MassARRAY® iPLEX platform, which is based on MALDI-TOF (matrix-assisted laser desorption/ionization time-of-flight) mass spectrometry*
Data source location	*Belgorod, Russia*
Data accessibility	*The data is available with this article*

**Table:** 

**Value of the data** • The genetic variants in *LIN28B* gene may play a role in age at menarche. • The data on the allele, genotype and haplotype frequencies are important because they contribute to understanding genetic structure of populations. • The data can be used in research of a genetic basis of age at menarche and menarche-associated multifactorial diseases (obesity, breast cancer, osteoporosis, uterine leiomyoma, endometriosis, preeclampsia and others) in various populations.

## 1 Data

**Fig. 1:**
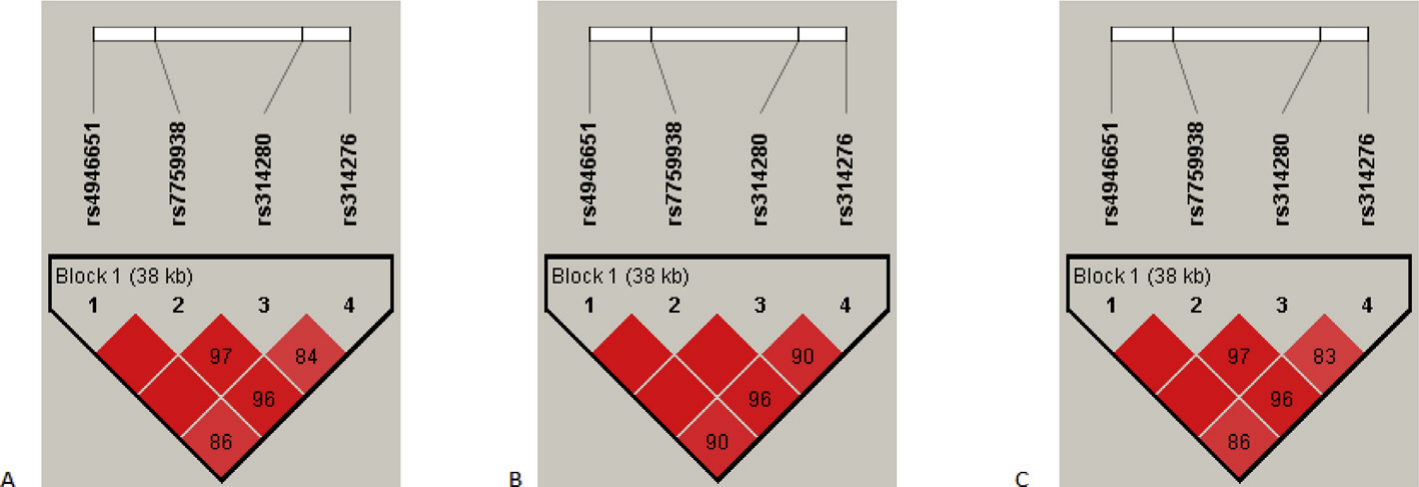
The structure of linkage disequilibrium of rs4946651, rs7759938, rs314280 and rs314276 in the *LIN28B* gene in the sample of Russian women. Linkage disequilibrium was measured by Lewontin's coefficient D′. The dark red (D′ = 1) indicates that there exists strong pairwise LD between SNPs. A) All sample set. B) Early age at menarche (<12 years). C) Average age at menarche (12–14 years).

The dataset represents the raw data (supplementary Table), frequencies of alleles, genotypes and haplotypes for single nucleotide polymorphisms (SNPs) rs4946651, rs7759938, rs314280 and rs314276 of the *LIN28B* gene associated with age at menarche in previously published genome-wide and candidate gene association studies https://www.ncbi.nlm.nih.gov/pmc/articles/PMC5738205/, [1], [2], [3], [4], [5], [6], [7]. The data were divided into three groups according to the age at menarche (AAM) of the participants: the whole sample, the early age at menarche (<12 years), and the average age at menarche (12–14 years). The frequencies of the alleles, genotypes and haplotypes are presented in https://www.ncbi.nlm.nih.gov/pmc/articles/PMC5738205/table/t0005/, Table 1 and https://www.ncbi.nlm.nih.gov/pmc/articles/PMC5738205/table/t0010/, Table 2 respectively. The structure of linkage disequilibrium of rs4946651, rs7759938, rs314280 and rs314276 in *LIN28B* gene is shown in https://www.ncbi.nlm.nih.gov/pmc/articles/PMC5738205/figure/f0005/, Fig. 1.

**Table 1:** The frequencies of alleles and genotypes for single nucleotide polymorphisms (SNPs) rs4946651, rs7759938, rs314280 and rs314276 of the *LIN28B* gene in the sample of Russian women.

SNP genotype or allele	All (n = 674)		Age at menarche				
			Mean, years	early (<12 yrs) (n = 66)		average (12–14 yrs) (n = 579)	
	n	frequency		n	frequency	n	frequency
rs4946651							
AA	120	0.1780	12.67 ± 1.00	8	0.1212	108	0.1865
GA	333	0.4941	12.65 ± 1.09	35	0.5303	282	0.4870
GG	221	0.3279	12.56 ± 1.03	23	0.3485	189	0.3265
A	573	0.4251	–	51	0.3864	498	0.4301
G	775	0.5749	–	81	0.6136	660	0.5699
rs7759938							
CC	52	0.0772	12.73 ± 1.12	4	0.0606	45	0.0777
TC	298	0.4421	12.67 ± 1.07	28	0.4242	255	0.4404
TT	324	0.4807	12.56 ± 1.03	34	0.5152	279	0.4819
C	402	0.2982	–	36	0.2727	345	0.2979
T	946	0.7018	–	96	0.7273	813	0.7021
rs314280							
TT	109	0.1617	12.68 ± 1.01	8	0.1212	97	0.1675
CT	344	0.5104	12.65 ± 1.09	35	0.5303	293	0.5060
CC	221	0.3279	12.56 ± 1.03	23	0.3485	189	0.3265
T	562	0.4169	–	51	0.3864	487	0.4206
C	786	0.5831	–	81	0.6136	671	0.5794
rs314276							
AA	63	0.0935	12.68 ± 1.10	6	0.0909	54	0.0933
CA	300	0.4451	12.71 ± 1.07	25	0.3788	259	0.4473
CC	311	0.4614	12.53 ± 1.02	35	0.5303	266	0.4594
A	426	0.3160	–	37	0.2803	367	0.3169
C	922	0.6840	–	95	0.7197	791	0.6831

**Table 2:** The frequencies of haplotypes for single nucleotide polymorphisms (SNPs) rs4946651, rs7759938, rs314280 and rs314276 of the *LIN28B* gene in the sample of Russian women.

Haplotype (rs4946651, rs7759938, rs314280 and rs314276	All (n = 674), frequency	Age at menarche	
		early (<12 yrs) (n = 66), frequency	average (12–14 yrs) (n = 579), frequency
ACTA	0.287	0.265	0.287
GTCA	0.024	0.015	0.024
ATTC	0.122	0.114	0.126
GTCC	0.551	0.598	0.547

## 2 Experimental design, materials, and methods

### 2.1 Subjects

The recruitment of the participants was carried out through the Perinatal Centre of the Belgorod Regional Clinical Hospital of St. Joasaph during 2008–2013. All participants were unrelated women of Russian descent (self-declared) living in Central Russia [8]. The following exclusion criteria were adopted: non-Russian descent, a birthplace outside of Central Russia, malignant tumors of a small pelvis and breast, benign tumors and hyperplastic disorders of the reproductive organs in women (leiomyoma, endometriosis, and endometrial hyperplasia), chronic severe diseases of the vital organs (heart, respiratory or renal failure), severe autoimmune diseases. The research protocol was approved by the Regional Ethics Committee of Belgorod State University. Written informed consent for participation was obtained from all individuals enrolled in the research.

The information about AAM was obtained using the questionnaire. AAM was defined as age (full years) of first menses. Each participant was asked a question: “How old were you when you had the first menses?” Women with AAM ≥18 years (n = 4) or women who refused to answer (n = 13) were excluded from the research. In total, 674 females participated in the research.

### 2.2 Blood sample collection and DNA handling

The phlebotomy was performed by a certified nurse. Five milliliters of blood was taken from the ulnar vein into a plastic vial (Vacutainer®) with 0.5M EDTA solution (рН = 8.0). Extraction of lymphocyte DNA was done by standard phenol-chloroform technique and quantified by Nanodrop 2000 spectrophotometer (Thermo Scientific, Inc.). Only samples with А260/А280 = 1.7–2.0 were used for the analysis.

### 2.3 SNP selection

The 4 SNPs in the *LIN28B* gene (rs4946651, rs7759938, rs314280 and rs314276) were selected for the analysis based on the following criteria [9], [10]: 1) Previously reported associations with AAM and phenotypes, which share metabolic pathways with menarche (e.g., obesity, anthropometric characteristics, vitamin D metabolism, etc.), 2) Regulatory potential (regSNP), 3) Effect on gene expression (eSNP), 4) Tag value (tagSNP) and 5) MAF > 5%.

The selected polymorphic loci have functional significance: all SNPs appear to have a significant regulatory potential (Table 3) (determined using the online tools HaploReg, v4.1 update 05.11.2015, https://pubs.broadinstitute.org/mammals/haploreg/haploreg.php) and to influence gene expression level (Table 4) (determined using the GTExportal data, http://www.gtexportal.org/).

**Table 3:** Regulatory effects of the 4 SNPs of the LIN28B gene (HaploReg, v4.1, update 05.11.2015) (https://pubs.broadinstitute.org/mammals/haploreg/haploreg.php).

pos (hg38)	variant	Ref	Alt	AFR	AMR	ASN	EUR	GERP	SiPhy	Promoter	Enhancer	DNAse	Proteins	Motifs	NHGRI/EBI	GRASP QTL	Selected eQTL	GENCODE	dbSNP
				freq	freq	freq	freq	cons	cons	histone marks	histone marks		bound	changed	GWAS hits	hits	hits	genes	func annot
104921635	rs4946651	A	G	0.18	0.63	0.70	0.52										1 hit	15kb 3′ of LINC00577	
104931079	rs7759938	C	T	0.37	0.72	0.70	0.65				ESC, IPSC				6 hits	2 hits		5.2kb 3′ of LINC00577	
104952962	rs314280	A	G	0.18	0.62	0.70	0.52			6 tissues	4 tissues	11 tissues	5 bound proteins		1 hit		1 hit	4.1kb 5′ of LIN28B	
104960124	rs314276	A	C	0.53	0.67	0.70	0.64			5 tissues	IPSC			HNF1,OTX,Pou2f2	2 hits	11 hits		LIN28B	intronic

**Table 4:** The cis-eQTL values of the 4 SNPs of the LIN2B gene (according to Genotype-Tissue Expression (GTEx) (http://www.gtexportal.org/)).

SNP	Gene expression	Allele ref	Allele alt	Effect Size (β)	P-Value	Tissue
rs4946651	*LIN28B*	A	G	−0.40	7.6х10^-8^	Pituitary
	*LINC00577*			0.58	0.0000016	Brain - Cortex
	*LINC00577*			0.48	0.0000022	Brain - Putamen (basal ganglia)
rs7759938	*LIN28B*	C	T	−0.50	1.3х10^-11^	Pituitary
rs314280	*LIN28B*	A	G	−0.40	7.6х10^-8^	Pituitary
	*LINC00577*			0.58	0.0000016	Brain - Cortex
	*LINC00577*			0.48	0.0000022	Brain - Putamen (basal ganglia)
rs314276	*LIN28B*	A	C	−0.50	9.4х10^-12^	Pituitary

### 2.4 SNP genotyping

DNA samples were genotyped using the Sequenom MassARRAY® iPLEX platform at the Centre of Genomic Sciences (University of Hong Kong). The procedure for DNA sample preparation and data quality control are described elsewhere [10].

### 2.5 Statistical analysis

The correspondence of the SNPs to the Hardy-Weinberg equilibrium was checked using the chi-square test. No significant differences in allele frequencies between the group with the early age at menarche (<12 years) and group with the average age at menarche (12–14 years) (p > 0.05) were revealed. The Haploview version 4.2 software (https://www.broadinstitute.org/haploview/haploview) was used to quantify the linkage disequilibrium (LD) between rs4946651, rs7759938, rs314280 and rs314276 in *LIN28B* gene. Haplotype frequencies were determined using the EM algorithm. The LD block structure was defined using the Solid Spine of the LD algorithm [11] provided by the Haploview 4.2. The degree of genetic linkage between the 4 SNPs in each groups was estimated as Lewontin's coefficient D′, where no color (D′ = 0) indicates that LD is weak or nonexistent and the dark red (D′ = 1) indicates that there exists strong pairwise LD between SNPs (Fig. 1).
